# Challenges in Diagnosing Psoriatic Arthritis in Primary Care: A Meta-Ethnographic Study

**DOI:** 10.7759/cureus.49443

**Published:** 2023-11-26

**Authors:** Ryuichi Ohta, Chiaki Sano

**Affiliations:** 1 Communiy Care, Unnan City Hospital, Unnan, JPN; 2 Community Medicine Management, Shimane University Faculty of Medicine, Izumo, JPN

**Keywords:** interdisciplinary collaboration, qualitative synthesis, disease management, rheumatology, early detection, clinical manifestations, diagnostic challenges, diagnosis, primary care, psoriatic arthritis

## Abstract

Psoriatic arthritis (PsA) is a complex and debilitating chronic inflammatory joint disorder that is often associated with psoriasis and presents significant challenges in its early diagnosis and management. Therefore, this study aimed to investigate the diagnostic intricacies of PsA in primary care settings to shed light on the prevalence, barriers, and implications of delayed diagnosis. To achieve our research objectives, we conducted a qualitative synthesis using the meta-ethnographic method, which is a robust approach for synthesizing qualitative data. We systematically searched the PubMed, Web of Science, and Embase databases for relevant articles using predefined search terms such as "psoriatic arthritis," "diagnosis," and "primary care." The inclusion criteria were narrative articles in English that provided insights into the diagnostic challenges of PsA in primary care. Conference presentations, original articles, and duplicate articles were excluded. Our analysis revealed four key themes that elucidated the multifaceted nature of PsA diagnosis in primary care: (1) a variety of initial and non-specific symptoms, highlighting the diverse clinical presentations that can mimic other conditions; (2) the lack of experience with PsA among primary care professionals, underscoring the importance of education and training; (3) the lack of skin lesions, which can complicate diagnosis when not present; and (4) a delay in diagnosis, with potentially severe consequences for patients' health and quality of life. This study highlights the challenges inherent in the diagnosis of PsA in primary care settings. The multifaceted nature of the disease, coupled with limited experience among primary care providers, often results in delayed diagnosis and subsequent treatment initiation. Early recognition and intervention are pivotal for optimizing patient outcomes. Addressing these challenges necessitates a comprehensive approach involving heightened clinical suspicion, continuous medical education, interdisciplinary collaboration, and utilization of standardized diagnostic criteria. Collaboration between primary care physicians and specialists is crucial for enhancing the accuracy and timeliness of PsA diagnosis and ultimately improving patient well-being and quality of life.

## Introduction and background

Psoriatic arthritis (PsA) is a chronic inflammatory joint disorder intrinsically linked to psoriasis that presents physical and psychological challenges for the affected patients [1]. Predominantly diagnosed and managed by primary care physicians and rheumatologists, the broad spectrum of symptoms of the disease, some of which overlap with other arthritic conditions, makes it a particularly complex ailment to identify [2].

Recent epidemiological research has shed light on the prevalence of PsA in primary care settings, painting a concerning picture. Studies have suggested that the prevalence of PsA oscillates between 7% and 13% [3]. To add nuance to these statistics, another study found that among primary care psoriasis patients, 3.2% also had PsA, which increased to 4.6% when including those with enthesitis [4]. Another alarming finding is that one in four patients with psoriasis may be battling PsA concurrently [5].

These numbers emphasize the importance of primary care physicians maintaining heightened vigilance. Diverse manifestations of the disease, from dactylitis to enthesitis, can be misleading, especially when they mirror the symptoms of ailments such as osteoarthritis or rheumatoid arthritis [6].

Effective management of PsA requires a holistic approach. Pharmacologically, non-steroidal anti-inflammatory drugs commonly offer initial relief, with more severe cases benefiting from disease-modifying antirheumatic drugs and biological agents that target specific immune responses [7]. However, medication is only one part of this puzzle. Physical therapy and lifestyle adaptations are equally important in therapeutic regimens [8]. Guided exercises can help restore mobility, reduce stiffness, fortify muscle strength, and prevent further joint deterioration. Embracing lifestyle changes, such as a balanced diet and low-impact activities, can also significantly manage the disease [9].

The crux of effective PsA management lies in early detection and intervention. Delays or misdiagnoses can result in irreversible joint damage and diminished quality of life [6]. Beyond the tangible physical repercussions, the psychological burden of living with undiagnosed or improperly managed PsA, which is often characterized by anxiety and depression, cannot be overlooked [10].

This study aimed to highlight the intricacies of diagnosing PsA in primary care. By understanding the hurdles, we aimed to create refined diagnostic protocols. We envision overcoming these challenges by combining clinical expertise with advanced diagnostic tools and patient histories. The ultimate goal is to ensure that patients receive timely and appropriate care, facilitating a life defined not by limitations, but by possibilities.

## Review

Methodology

We performed a qualitative synthesis using the meta-ethnography method, which was used to synthesize the qualitative data [11-13]. Original articles on meta-ethnography suggest that this method can also be used to synthesize qualitative data in scientific papers. Originally, meta-ethnography was developed to synthesize all qualitative studies that clarified the deep parts of the real world. In clinical medicine, the various context-based experiences and knowledge of specialists have been summarized in narrative reviews and original article discussions. These experiences and the wisdom of narrative reviews and discussions cannot be synthesized using quantitative methods [11-13]. Meta-ethnography can be a helpful methodology for synthesizing such data. This process can also be applied to the qualitative synthesis of narrative review articles [11-13]. We used a meta-ethnography to synthesize qualitative data on the difficulty of diagnosing PsA in primary care.

Based on the research question, we decided on search terms using the framework of population, types of study, and included content. Our search terms were: “psoriatic arthritis,” “diagnosis,” and “primary care.” We searched for the relevant articles on PubMed, Web of Science, and Embase and collected the related articles comprehensively. The search strategy used was as follows: “psoriatic arthritis” AND “diagnosis” AND “primary care.”

Study selection

The inclusion and exclusion criteria are presented in Table 1.

**Table 1:** Inclusion and exclusion criteria

	Inclusion Criteria	Exclusion Criteria
Population	Psoriatic arthritis	Other diseases
Types of study	Original articles, reviews	Non-empirical studies (editorial, news, and conference papers)
Included content	Diagnosis of psoriatic arthritis in primary care	
Other	Abstract available, full text available in English	Abstract not available, full text not available in English

Narrative articles were included in the meta-analysis, while conference presentations and duplicate articles were excluded.

Data extraction

The first investigator (R.O.) independently conducted literature searches and data extraction and extracted information regarding the difficulty of diagnosing PsA in primary care from the selected articles using a purpose-designed data extraction form. The second investigator (C.S.) checked the extracted content, which was synthesized using meta-ethnography.

To ensure credibility, the investigators discussed the extracted data. Any discrepancies were resolved through discussion. Articles regarding the difficulty of diagnosing PsA in primary care without clear descriptions of the aims, participants, or outcomes were excluded (Table 1). In the case of difficulty in categorizing and extracting data, the investigators discussed the contents until an agreement was reached.

Analysis

A qualitative synthesis conducted via meta-ethnography was performed using the following eight steps: getting started, deciding what was relevant to our initial interest, reading the studies, determining how the studies were related, rereading the studies, translating the studies into one another, synthesizing the translations, and detailing the synthesis [11-13]. The first step involved searching for related articles using search engines. To determine what was relevant to our initial interests, the first investigator selected the reviews to be included in the meta-ethnography by reading the abstracts and checking for concordance with the inclusion criteria. Subsequently, the first investigator repeatedly read all the selected reviews and extracted the sections relevant to the difficulty in diagnosing PsA in primary care. The vague sections were discussed with a second investigator to determine their inclusion in the analysis. Studies were then translated into one another by inductively coding the extracted content. We thematically synthesized the concepts and themes that appeared in each review for translation synthesis. For triangulation, the concepts and themes were discussed among the researchers and analyzed iteratively during the review period after the completion of a tentative analysis of reviews for theoretical saturation.

Results

**Figure 1:**
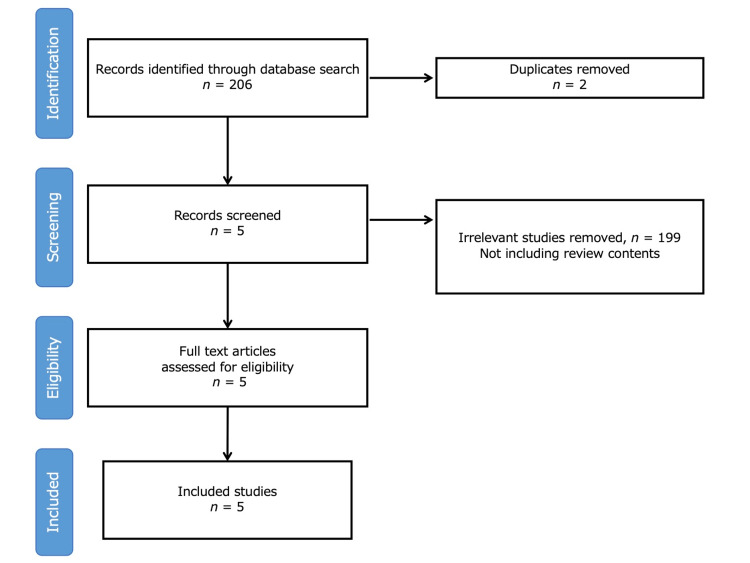
Selection flowchart of the included reviews

Of the 206 studies analyzed, two were excluded because of duplication. After reviewing the abstracts, 199 studies were excluded because they did not include reviewing content. Ultimately, five articles were included in the final analysis based on the difficulty of diagnosing PsA in primary care (Figure 1).

The included articles are the following (Table 2).

**Table 2:** The demographics of the included articles

Reference number	Publication year	First author	Title
[14]	2022	Chang J	Utilization of the psoriasis epidemiology screening tool (PEST): a risk stratification strategy for early referral of psoriatic arthritis patients to minimize irreversible erosive joint damage
[15]	2021	Dures E	Diagnosis and initial management in psoriatic arthritis: a qualitative study with patients
[16]	2016	Coates LC	Comparison of screening questionnaires to identify psoriatic arthritis in a primary-care population: a cross-sectional study
[17]	2021	Gratacós J	A 12-point recommendation framework to support advancement of the multidisciplinary care of psoriatic arthritis: a call to action
[18]	2021	Guillen Astete CA	Delay and diagnostic pathway of patients with psoriatic arthritis in Spain

Through meta-ethnography, four themes were identified (Table 3).

**Table 3:** Themes and explanations PsA: psoriatic arthritis

Theme	Explanation
Variety of initial and non-specific symptoms	Diagnosing PsA in primary care is challenging because initial symptoms of PsA can vary and be not specific.
Lack of experience with PsA	The lack of experience is a limit for the diagnosis of PsA.
The lack of skin lesions	The lack of skin lesions can make the diagnosis of PsA difficult.
A delay in diagnosis	A delay in diagnosis can be a critical issue in primary care.

The broad spectrum of initial and non-specific symptoms implies that patients may present with varied clinical manifestations, making it difficult for primary care physicians to identify PsA as the cause. Moreover, some primary care professionals' rare encounters or unfamiliarity with PsA further exacerbate diagnostic challenges. Although PsA is often associated with skin lesions, the absence of such lesions in some patients muddles the diagnostic picture, potentially leading to misdiagnosis or overlooking the possibility of PsA. Finally, any delay in diagnosis, irrespective of the cause, can have significant implications for patient health and disease management. These themes collectively underscore the multifaceted complexities that primary care professionals encounter when diagnosing PsA.

Variety of Initial and Non-specific Symptoms

PsA presents a peculiar diagnostic conundrum in primary care owing to its myriad initial symptoms. Patients may exhibit a wide array of clinical manifestations that mimic other conditions or are so subtle that they are overlooked. Such varying presentation and disease severity were observed in one of the included articles, which resulted in delayed treatment with disease-modifying antirheumatic drugs [14]. For instance, joint pain, a common symptom of PsA, can be easily confused with other forms of arthritis or unrelated musculoskeletal disorders. Another included article showed that participants’ own beliefs as to the causes of their symptoms, such as excessive physical exertion, stress, aging, childhood physical activity, sports injuries, and post-pregnancy or menopause effects, and the resulting view that intervention was not necessary contributed to delays in seeking professional consultation [15]. Furthermore, other systemic manifestations, such as fatigue or reduced range of motion, may be attributed to different causes altogether. An included article showed that our estimate of 18.1% was likely to be overestimated, as people with musculoskeletal symptoms are presumably more likely to respond and attend examinations, creating a selection bias [16]. This vast and overlapping symptomatology not only clouds the clinical picture but also demands a high index of suspicion from primary care providers.

Lack of Experience With PsA

The limited exposure of some primary care professionals to PsA has accentuated the diagnostic hurdles. Given the rarity of the condition and the possibility that many cases remain undiagnosed, many primary care practitioners may require more clinical encounters with PsA and consequently are unfamiliar with its presentation. The included studies show that in the absence of a definitive test, rheumatologists rely on their clinical expertise and patient examination (medical history, physical examination, blood tests, MRI, and radiography) to diagnose PsA [14,15]. However, interpreting symptoms was often difficult and was further complicated when, for example, blood test results were "normal" [15]. This lack of experience can result in misdiagnosis, inappropriate referrals, or unnecessary investigations, all of which can delay appropriate treatment. Continuous medical education and training may help bridge this gap, ensuring that primary care professionals are equipped to recognize and manage PsA effectively.

Lack of Skin Lesions

Although psoriasis, characterized by skin lesions, is a hallmark of PsA, its absence in some patients makes the diagnostic journey even more complicated. When encountering a patient with joint issues, primary care professionals may prioritize more common causes and overlook PsA if characteristic skin lesions are not present [17,18]. According to the included article, most patients diagnosed with PsA present with psoriasis first, whereas 20% present with PsA first and <10% experience both sets of symptoms simultaneously. As symptoms of psoriasis are generally reversible, whereas joint damage from PsA is not, prioritizing early diagnosis and treatment of PsA is especially crucial [17]. This is particularly pertinent because skin manifestations do not always precede joint symptoms. Thus, a patient may present with arthritic symptoms before any skin lesions become apparent or may never develop noticeable skin manifestations. This theme emphasizes the importance of not relying solely on dermatological signs when considering a PsA diagnosis.

Delay in Diagnosis

A timely diagnosis of PsA is paramount, given the progressive nature of the disease. Any delay can lead to irreversible joint damage, a decreased quality of life, and other complications. In primary care, where first contact with patients usually occurs, a delay can be attributed to the previously mentioned themes: the vast range of initial symptoms, lack of experience with the disease, and absence of skin lesions [17]. One of the included articles reported that six-month diagnosis and treatment delays resulted in an increased risk of peripheral joint erosion and poorer patient-reported outcomes [17]. Long delays (≥1 year) between symptom onset and diagnosis predicted worse physical functioning later in the disease course. [17]. Another included article discussed points of early referral centered around the relationship between psoriasis and PsA that could promote early diagnosis, although there is no data on a reduction in referral time [18]. Each day without appropriate intervention can exacerbate the patient's condition, emphasizing the need for early recognition and management. This theme culminates in the challenges faced and underscores the repercussions of not addressing diagnostic barriers.

Discussion

This meta-ethnography highlighted the multifaceted challenges primary care professionals face when diagnosing PsA. The variety of initial symptoms, lack of experience among healthcare providers, absence of skin lesions, and delays in diagnosis are significant barriers to timely and accurate disease identification.

A variety of initial symptoms can develop in patients with PsA in primary care and should be effectively taught to primary care physicians. The diverse manifestations of PsA underscore the diagnostic challenges in primary care settings [19]. For instance, joint pain, commonly associated with PsA, has several differential diagnoses ranging from other types of arthritis to noninflammatory musculoskeletal disorders [19,20]. This is further complicated by patients attributing their symptoms to various causes such as physical strain, psychological stress, or aging [15,21]. Presentation heterogeneity can significantly delay treatment initiation, particularly for targeted therapies, such as disease-modifying antirheumatic drugs [14]. Overlap with other conditions such as rheumatoid arthritis and polymyalgia rheumatica also underscores the importance of clinical suspicion, particularly among older patients [13,19,20]. As the aforementioned evidence indicates, there is potential for selection bias when determining the prevalence of musculoskeletal symptoms, which further emphasizes the challenges of PsA recognition in a sea of musculoskeletal complaints [16,22].

The lack of experience with PsA among primary care physicians impinges on the diagnosis and management of PsA in primary care. Although the diagnostic approach for PsA is based on clinical expertise, physical examination, and imaging techniques [14], many primary care professionals may need to gain experience with the disease [23]. Challenges faced by GPs in interpreting symptoms, especially when blood tests are normal, point to the complexities of the clinical picture of PsA [15]. The intricacies of a diagnosis that relies on a combination of clinical, radiological, and sometimes serological factors emphasize the need for further training and exposure to PsA presentations in primary care settings.

The lack of skin lesions is not rare in the initial presentation of PsA and should be taught to primary care physicians, and collaboration between primary care physicians and rheumatologists should be enhanced [24]. The dissociation between skin and joint symptoms in some patients with PsA poses a diagnostic dilemma [25]. As highlighted, while most patients with PsA present with psoriasis before arthritis manifestation, a significant proportion may first display joint symptoms [17]. Because joint damage is often irreversible, an early and accurate diagnosis is imperative. Furthermore, the absence of skin lesions can lead primary care professionals to rule out PsA, emphasizing the importance of considering a broader clinical context and other potential indicators of the disease [24].

**Figure 2:**
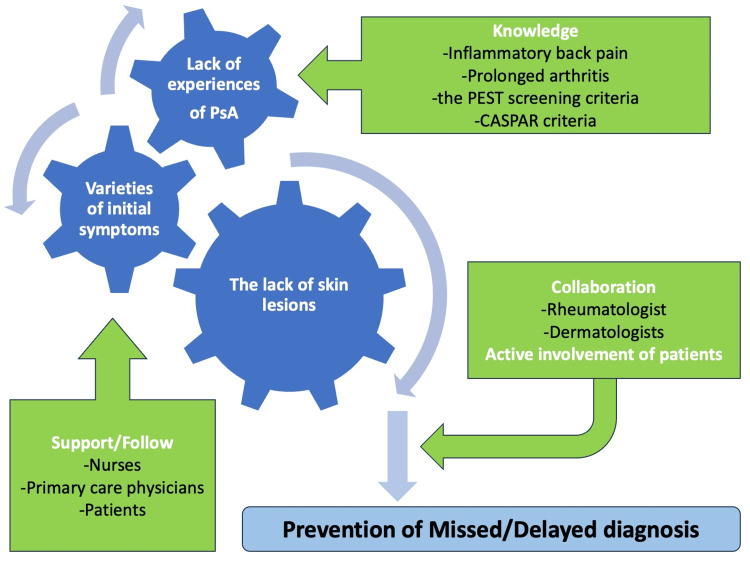
Comprehensive management of PsA in primary care CASPAR: Classification Criteria for Psoriatic Arthritis, PEST" Psoriasis Epidemiology Screening Tool, PsA: psoriatic arthritis

Consequently, a delay in diagnosis can occur owing to the aforementioned reasons, which should be approached comprehensively. Diagnostic delays in PsA have severe consequences. Evidence suggests that even a delay of six months can escalate the risk of joint erosion and detrimentally impact patient outcomes [17,26]. This implies that primary care professionals, often the first point of contact, play a crucial role in the early identification and management of PsA [27]. Primary care physicians should be educated more effectively regarding knowledge about inflammatory back pain and prolonged arthritis suggesting the possibility of PsA. Data showing no concrete reduction in referral times even after intensifying training indicate that more efficient diagnostic strategies, perhaps involving interdisciplinary collaboration, are required [18]. In an interdisciplinary collaboration, primary care physicians, nurses, and patients should discuss the patients' symptoms comprehensively. Furthermore, evidence points toward the use of criteria such as the Psoriasis Epidemiology Screening Tool (PEST) and the Classification Criteria for Psoriatic Arthritis (CASPAR) for a more accurate assessment of PsA [14,16]. Dialogue between primary care physicians and citizens can drive patients’ help-seeking behaviors with joint pain, leading to the effective management of PsA [28]. Through an effective understanding of PEST and CASPAR among primary care physicians, collaboration between primary care physicians, rheumatologists, and dermatologists can be facilitated. Given the complexities and overlapping presentations, employing standardized criteria can offer a more structured diagnostic approach (Figure 2).

This study had several limitations. First, it relied heavily on the qualitative meta-ethnography method. Although it is adept at synthesizing qualitative data, the findings may not represent quantifiable measures, making them less generalizable. Second, the selection of articles was limited to those available in PubMed, Web of Science, and Embase, which potentially overlooked relevant studies in other databases. Additionally, our focus on narrative reviews while excluding original articles and conference presentations may have led to missing insights and recent findings. The selection criteria limited the studies to those available in English and excluded valuable research from non-English sources.

Furthermore, any synthesis carries the risk of investigator bias, and despite our attempts to triangulate the data and resolve discrepancies among investigators, inherent biases in the extraction and interpretation of qualitative data could persist. Finally, although the identified themes offer a comprehensive understanding of the complexities of diagnosing PsA in primary care, other unexplored themes or nuances may need to be captured in our synthesis. Future research may benefit from a mixed methods approach that includes diverse data sources and broader search parameters to ensure a more exhaustive understanding.

## Conclusions

The intricacies of diagnosing PsA in primary care settings are evident. Drawing on the presented evidence, it is clear that a multifaceted approach integrating heightened clinical suspicion, continuing medical education, interdisciplinary collaboration, and standardized diagnostic criteria is pivotal for timely and accurate PsA diagnosis. Addressing these challenges will facilitate diagnosis and significantly impact patient outcomes by ensuring early and effective interventions.
